# PCR-Independent Detection of Bacterial Species-Specific 16S rRNA at 10 fM by a Pore-Blockage Sensor

**DOI:** 10.3390/bios6030037

**Published:** 2016-07-22

**Authors:** Leyla Esfandiari, Siqing Wang, Siqi Wang, Anisha Banda, Michael Lorenzini, Gayane Kocharyan, Harold G. Monbouquette, Jacob J. Schmidt

**Affiliations:** 1 Department of Bioengineering, University of California, Los Angeles, CA 90095, USA; esfandla@ucmail.uc.edu (L.E.); anisha.banda@gmail.com (A.B.); mhlorenzini@gmail.com (M.L.); gaiakocharyan@gmail.com (G.K.); 2 Department of Chemistry and Biochemistry, University of California, Los Angeles, CA 90095, USA; wangsiqing19940@ucla.edu; 3 Department of Chemical and Biomolecular Engineering, University of California, Los Angeles, CA 90095, USA; wangsiqi940103@ucla.edu

**Keywords:** nucleic acid detection, peptide nucleic acid (PNA), 16S rRNA, *E. coli* detection

## Abstract

A PCR-free, optics-free device is used for the detection of *Escherichia coli* (*E. coli*) 16S rRNA at 10 fM, which corresponds to ~100–1000 colony forming units/mL (CFU/mL) depending on cellular rRNA levels. The development of a rapid, sensitive, and cost-effective nucleic acid detection platform is sought for the detection of pathogenic microbes in food, water and body fluids. Since 16S rRNA sequences are species specific and are present at high copy number in viable cells, these nucleic acids offer an attractive target for microbial pathogen detection schemes. Here, target 16S rRNA of *E. coli* at 10 fM concentration was detected against a total RNA background using a conceptually simple approach based on electromechanical signal transduction, whereby a step change reduction in ionic current through a pore indicates blockage by an electrophoretically mobilized bead-peptide nucleic acid probe conjugate hybridized to target nucleic acid. We investigated the concentration detection limit for bacterial species-specific 16S rRNA at 1 pM to 1 fM and found a limit of detection of 10 fM for our device, which is consistent with our previous finding with single-stranded DNA of similar length. In addition, no false positive responses were obtained with control RNA and no false negatives with target 16S rRNA present down to the limit of detection (LOD) of 10 fM. Thus, this detection scheme shows promise for integration into portable, low-cost systems for rapid detection of pathogenic microbes in food, water and body fluids.

## 1. Introduction

Reportedly, 95% of deaths in the developing world are due to lack of access to proper diagnostics for infectious diseases [1]. Even in the developed world, most infectious disease diagnosis is accomplished by culturing methods that typically take days. Common pathogens, whose very presence *at any level* in body fluid is abnormal and is an indicator of infection include Group A *Streptococcus*, *Neisseria gonorrhoeae* (gonorrhea), *Chlamydia trachomatis* (chlamydia), influenza virus, and *Bordetella pertussis* (whooping cough) among many others. In recent years, demand for a binary (qualitative) test also has become apparent for the rapid screening of food for pathogens such as shiga-toxin producing *Escherichia coli* (*E. coli*) or *Listeria monocytogenes*. Clearly, there is strong motivation for development of sensitive, robust and portable molecular diagnostic devices that can give rapid yes/no results for the presence of important bacterial pathogens in food, water and body fluids.

Essentially, there exist two options for pathogen diagnosis by molecular means: immunoassays and detection of pathogen-specific nucleic acid (NA). However, many of the existing platforms for NA detection at sufficiently low concentration require amplification by PCR, fluorescent or enzymatic labels, and expensive instrumentation [2,3]. It is believed that the most successful of these detection modalities for point-of-care (POC) applications ultimately will be NA amplification-free, will entail electrical/electrochemical signal transduction, and will be compatible with a microfluidic device platform [4,5,6]. Although fluorescence-based schemes improve sensitivity, the integration of optics into a device generally adds bulk, complexity, and cost. Also, although appealing alternatives to PCR for NA amplification have emerged, including some isothermal schemes that can be accomplished in minutes, they all, like PCR, require expensive reagents (e.g., primers) and reactions that must be controlled [7,8,9,10,11]. Thus, avoidance of NA amplification remains an important objective.

Recently, NA amplification-free electrochemical methods were demonstrated for the detection of specific RNA sequences in urine [12] and saliva [13]. In saliva, an impressive 0.4 fM limit of detection (LOD) was claimed using a detection scheme based on a novel oligonucleotide hairpin capture probe immobilized on an electrode with a horseradish peroxidase-labeled antibody for amperometric signal amplification [14]. Other clever, low LOD, NA amplification-free approaches for electrochemical DNA detection have been reported, including a conductometric method utilizing Au nanoparticles and silver development [15] and another scheme involving silver deposition with the signal arising from an anodic stripping current of the deposited silver [16]. These electrochemical methods for NA sequence detection are attractive for the avoidance of NA amplification, compatibility with microfluidic devices, and amenability to implementation in a compact array format. Yet, ideally, this same or better LOD would be achieved without the use of reporter probes and special reagents other than the capture probe oligonucleotides.

When detection schemes requiring NA amplification, optics, and reagents other than the capture probe are removed from consideration, the number of reported technologies is very small and includes cantilever-based methods and nanotube- or nanowire-based approaches [17] The cantilever-based approach entails the detection of a decrease in resonance frequency of vibration upon target NA binding to probes on the cantilever. The resonance frequency typically is monitored using a laser [18], and the system is subject to problems arising from viscous damping in liquids [17], but a recent millimeter-scale design circumvents this issue and resonance frequency changes are monitored with an impedance analyzer [19]. Nanotube/nanowire-based detection schemes have their foundation in the field effect transistor concept, and NA binding is sensed by a change in current-voltage (i–V) response. A recent report describes a polypyrrole nanowire system that provides an impressive LOD of 0.1 fM [20]. As these and other technologies are tested further in molecular diagnostic applications, clearer advantages and disadvantages, likely application-specific, will emerge; however, it would be desirable to transduce the probe to target NA hybridization with simpler, low-cost electronics.

Due to species-specific sequence variations, 16S ribosomal RNA (or the corresponding gene) commonly has been used as a pathogen-specific target in numerous detection schemes [21,22,23]; and we hypothesized that our previously described, low-cost device for sequence-specific nucleic acid detection may provide a platform for detection of pathogenic bacterial 16S rRNA. Our technology is based on the observed step change reduction in ionic current through a pore when blocked by a peptide nucleic acid-functionalized bead that has acquired electrophoretic mobility upon target NA hybridization [24]. We demonstrated the operation of our device for PCR-free detection of longer DNA targets (1613 bases) with limit of detection (LOD) down to 10 fM in the presence of a high concentration of non-specific DNA to simulate the genomic background of a realistic biological application [25]. Since 16S rRNA is close in size (~1500 bases) to that detected in our previous work and it has high copy number (6700–71,000 per viable cell) [26], it may prove possible to detect 16S rRNA at a level corresponding to a low viable bacterial cell concentration (colony forming units/mL (CFU/mL)) with our device.

Following a study by Stender et al. [27], we targeted a specific sequence within the 16S rRNA of an easily cultured non-pathogenic laboratory *E. coli* strain, (ATCC 25922). This strain offers an attractive test system for our technology since its genome has been sequenced and studied thoroughly. Also, an optimal probe sequence complementary to its 16S rRNA has been extensively tested by other groups [28,29,30]. As reported here, we studied the performance of our detector for species-specific *E. coli* 16S rRNA by extracting the total RNA from viable cells and investigating the detection limit of the sensor in terms of 16S rRNA concentration. Control experiments were conducted with the total RNA from two other bacterial species, *Pseudomonas fluorescens* (ATCC 13525) and *Pseudomonas putida* (ATCC 12633), whose 16S rRNA genes are closely related to those of the target *E. coli* bacterium. For a positive control, we used a 15-base universal PNA probe sequence, which is complementary to all three bacterial 16S rRNAs and has been used by other groups for detecting *E. coli* [31].

## 2. Materials and Methods

### 2.1. Chemical and Biological Materials

All chemicals were purchased from Sigma-Aldrich (St. Louis, MO, USA) and used as received unless otherwise noted. Carboxylic acid-functionalized, 3-µm-diameter polystyrene microspheres were purchased from Polysciences, Inc. (Warrington, PA, USA). Peptide nucleic acid (PNA) was purchased from Bio-Synthesis, Inc. (Lewisville, TX, USA) as HPLC-purified and lyophilized powders. The PNA probe for detecting *E. coli* 16S rRNA was NH_2_-(CH_2_CH_2_O)_12_- CTC CTT CCC TCA TTT CA [27]. For positive control experiments, the universal PNA probe NH_2_-(CH_2_CH_2_O)_12_- CTG CCT CCC GTA GGA was used [31]. Methoxy-polyethylene glycol amine, CH_3_O-(CH_2_CH_2_O)_3_-NH_2_ (MW 350) was obtained from Nanocs, Inc. (New York, NY, USA). Pre-pulled borosilicate micropipettes with 2 µm inside tip diameter were purchased from World Precision Instruments, Inc. (Sarasota, FL, USA). All bacteria: *E. coli* (ATCC 25922), *Pseudomonas fluorescens* (ATCC 13525), and *Pseudomonas putida* (ATCC 12633), and culture medium ingredients, soy agar and nutrient agar were purchased from ATCC, Inc. (Manassas, VA, USA). PureLink RNA Mini Kit, PureLink DNase Set, RNase free water, RNase-free pipette tips, RNase-away reagent and RNase-free microfuge tubes were purchased from Invitrogen Life Technologies (Grand Island, NY, USA).

### 2.2. Probe Coupling to Microspheres

Fifty microliters of 3-µm-diameter, carboxylic acid-functionalized polystyrene microspheres at 1.69 × 10^9^/mL were washed three times with MES buffer (60 mM, 2-(N-morpholino)ethanesulfonic acid, pH 5.5). The diameter (determined by dynamic light scattering (DLS)) and zeta potential (described below) of the beads before conjugation was measured with a Zetasizer Nano-ZS (Malvern Instruments) and found to be 3720 nm and −87 mV, respectively. After each wash, the microspheres were centrifuged at 14,000 rpm for 15 min; after the third wash and centrifugation, the beads were resuspended in 0.6 mL coupling buffer (100 mM, 1-[3-(dimethylamine)propyl]-3-ethylcarbodiimide (EDC) in MES buffer) and incubated at 50 °C for 45 min. Ten nanomoles of amine-functionalized PNA detection probes were added to the coupling buffer and incubated with the beads at 50 °C for two hours. mPEG-amine (100 mM) was added to the reaction mixture and incubated at 50 °C for one hour to cap most remaining carboxylic acid groups on the bead surfaces so as to reduce aggregation and nonspecific binding of nucleic acids to the beads. Subsequently, 100 mM ethanolamine was added to the bead suspension to cap residual carboxyl groups, and the preparation was incubated at 50 °C for an additional hour. The same procedure was repeated for coupling the universal PNA probes to the beads. Finally, the beads were washed four times in 0.4 × SSC buffer (60 mM NaCl, 6 mM trisodium citrate, 0.1% Triton X-100, pH 8) and stored in PBS buffer (137 mM NaCl, 2.7 mM KCl, 10 mM Na_2_HPO_4_, 2 mM KH_2_PO_4_, pH 7.4) at 4 °C. Prior to hybridization, the zeta potentials of PNA-bead batches were measured in 1 mM KCl, 10 mM HEPES (pH 7.0) to confirm the near charge neutrality of the beads. The zeta potential after four washes for beads conjugated with *E. coli*-specific PNA and capped with PEG and ethanolamine was −3.75 mV, and −3.27 mV for beads coupled with the universal PNA probes. At this low zeta potential, the beads tended to aggregate despite the PEG capping, which prevented meaningful diameter measurement by dynamic light scattering.

### 2.3. Sample Preparation

#### 2.3.1. Cell Culturing and Counting

Tryptic soy medium (15 g Tryptic Soy Broth in 500 mL water, pH 6.8) was prepared for culturing *E*. *coli* (ATCC 25922). Nutrient broth medium (4 g Nutrient Broth in 500 mL DI water, pH 6.8) was prepared for culturing *Pseudomonas putida* (ATCC 12633) and *Pseudomonas fluorescens* (ATCC 13525). After preparing the ATCC culture media, they were steam sterilized at 121 °C for 20 min. Bacterial culture plates for colony counting were prepared according to standard procedures (ATCC).

A starter culture was prepared by mixing lyophilized *E. coli* (ATCC 25922) with 1 mL culture tryptic soy medium, diluting that into 10 mL culture medium followed by incubation at 37 °C and 240 rpm until log phase was reached. Then, 1 mL of this starter culture was diluted into 250 mL culture medium and incubated at 37 °C and 240 rpm until log phase. To assess log phase, the optical density was measured at a wavelength of 600 nm (Genesys, Thermo Scientific) at 30 min intervals, and the culture was removed from the incubator at an optical density of 0.4–0.6. Similar preparative steps were repeated for the two control *Pseudomonas* strains in nutrient broth medium at 26 °C and 240 rpm. Log phase cells from the three different bacterial cultures were diluted serially into sterile saline, plated and incubated overnight in preparation for later colony counts. Simultaneously, 1 mL of original culture was prepared for RNA extraction by centrifugation at 25,000× *g* for 15 min at 4 °C. After removing the supernatant, cell pellets were immediately processed for total RNA extraction as described below.

#### 2.3.2. RNA Extraction and Purification

Total RNA extraction and purification was performed using the PureLink RNA Mini Kit and PureLink DNase Set, and the total extracted RNA was eluted in 100 μL RNase-free purified water. After RNA extraction from all bacterial dilutions, 5 µL of total RNA from each sample was measured for concentration and integrity using an Agilent 2100 Bioanalyzer.

### 2.4. Hybridization

Prior to hybridization, 2.1 × 10^6^ PNA-beads were washed twice with wash buffer (10 mM CAPSO, 0.2% (*v*/*v*) Tween 20, pH 10) and once with hybridization buffer (10 mM NaCl, 25 mM Tris-HCl, pH 7.0). Total RNA extracts from target and control bacteria were diluted serially to achieve the desired concentrations for device testing. RNA samples then were hybridized with PNA-beads functionalized with the target probe and the universal probe separately in 100 µL reaction volumes of hybridization buffer at 68 °C overnight.

### 2.5. Detection System and Electrical Measurements

**Figure 1:**
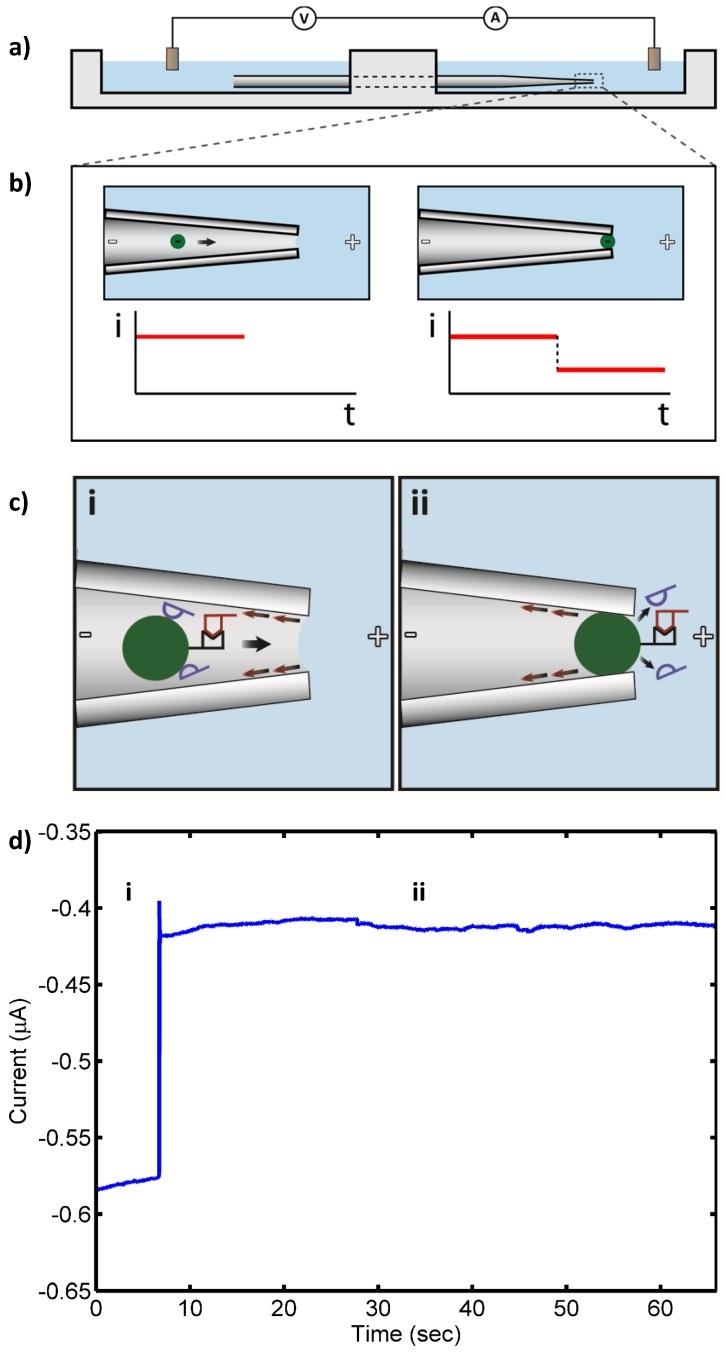
(**a**) Apparatus schematic. A micropipette drawn to a 2 µm diameter bridges two chambers filled with electrolyte. Negatively charged 3 µm diameter beads placed in the micropipette are electrophoretically drawn to the narrow end of the micropipette by an electric field applied by electrodes in each of the chambers; (**b**) When a bead reaches the narrow end of the micropipette, it blocks the current, which is measured by the electrodes; (**c**) Schematic of target 16S rRNA (10 pM, red) hybridized to PNA-bead conjugates in the drawn micropipette under applied electric field in the presence of nonspecifically bound, background RNA (blue): (i) open pore state as bead with bound RNA approaches the micropipette tip (“pore”); and (ii) blockade of the pore by the PNA-bead conjugate with bound RNA (blocked pore state). Nonspecifically bound RNA (blue) detaches from the bead in the strong electric field at the micropipette tip, while the specifically bound RNA remains, leading to a persistent blocked pore state; (**d**) Measured ionic current through the pore: (i) open pore current; and (ii) current blockade by PNA-bead conjugates with bound RNA.

After hybridization, samples were washed twice in wash buffer and resuspended in 100 µL of 1 mM KCl, 10 mM HEPES pH 7.0 prior to introduction into the device. Total RNA extracted from target and control bacteria incubated with the target PNA-beads and universal PNA-beads were injected into the large opening of the micropipette and a potential of 25 V was applied to direct the beads having bound NA to the micropipette tip, which serves at the “pore” in this incarnation of our system (Figure 1). More details of the detector, which consists of a micropipette connecting two buffer reservoirs, whose tip is drawn to an internal diameter of 2 µm, can be found in an earlier publication [24]. A Pt electrode is placed in each chamber to enable imposition of a constant potential difference. Current data was gathered on a PC (PCI 6052, National Instruments) with LabView software (National Instruments). The gathered data were processed using a MATLAB implementation of a fifth-order Butterworth 100 Hz lowpass filter. The device, including the micropipette and electrodes, was mounted on the stage of a Leica DMIRB inverted microscope for simultaneous microscopic observation. Device mounting and sample measurement required at least 10 min each.

## 3. Results

The experiments were designed to investigate the accurate detection of the target *E. coli* and establish the limit of detection (LOD) of our detector in terms of the concentration of 16S rRNA. To assess the LOD of our system, extracted total RNA samples from approximately 10^9^ CFU/mL were quantified and inspected using an Agilent Bioanalyzer 2100, serially diluted, and then introduced into our detector.

The Bioanalyzer was used to measure RNA concentration, size, and integrity number using an on-chip gel electrophoresis technique. The concentration of total RNA extracted from the target *E. coli* was 364 ng/µL. The percentage of 16S rRNA was 18.2% of total RNA, thus the concentration of 16S rRNA was 66.3 ng/μL. Using an approximate molecular weight of 1500-base ssRNA (480,909 g/mol), the molar concentration of 16S rRNA was calculated to be 137.8 nM. The Bioanalyzer measured the size of 16S rRNA to be 1494–1924 nucleotides. Also, it is essential to check the quality of the extracted RNA since RNA molecules are susceptible to degradation by RNAse present in the environment. The Bioanalyzer instrument measures the RNA integrity number based on evaluation of the 23S and 16S ribosomal ratios using an RNA integrity number algorithm. The integrity number of extracted total RNA from target *E. coli* was 9.2 (10 represents highest integrity and 0 represents lowest integrity), indicating that the extracted RNA was largely intact.

The same measurements and calculations were repeated for RNA extracted from the two control bacteria. For the control, *Pseudomonas putida* (*P. putida*, ATCC 12633), the total extracted RNA concentration was 449 ng/µL and the percentage of 16S rRNA was 25.7%, giving a 16S rRNA concentration of 115.4 ng/μL. The size of the 16S rRNA was reported to be 1260–1914 nucleotides, and the RNA “integrity number” was 10. The calculated 16S rRNA concentration was 239 nM.

The total extracted RNA concentration of the other control, *Pseudomonas fluorescens* (*P. fluorescens*, ATCC 13525), was 261 ng/µL and the percentage of 16S rRNA was 24.5%, giving a 16S rRNA concentration of 64 ng/μL. The 16S rRNA size was 1294–1984 nucleotides and the RNA “integrity number” was 10. We calculated the 16S rRNA concentration to be 133 nM. The data reported by the gel chip electrophoresis analysis of extracted total RNA from target and control bacteria is shown in the Supplementary Materials (Figure S1).

Following the RNA analysis, extracted total RNA samples were serially diluted, based on their 16S rRNA concentrations, to concentrations between 10 pM and 1 fM by dilution factors of 10. Diluted samples were then each hybridized separately with 2.1 × 10^6^ target and universal PNA-beads in high stringency conditions to reduce non-specific binding and enhance selectivity. These high stringency hybridization conditions were carefully chosen based on a study by Li et al. in which the same conditions were used for quantitative *Dechlorosoma suillum* rRNA detection by PNA molecular beacon assays [32]. After hybridization, in separate experiments, samples at each dilution were injected into the micropipette sensor apparatus and the electrical conductance measurements were performed. Table 1 summarizes the results of the experiments from 10 pM to 1 fM.

**Figure 2:**
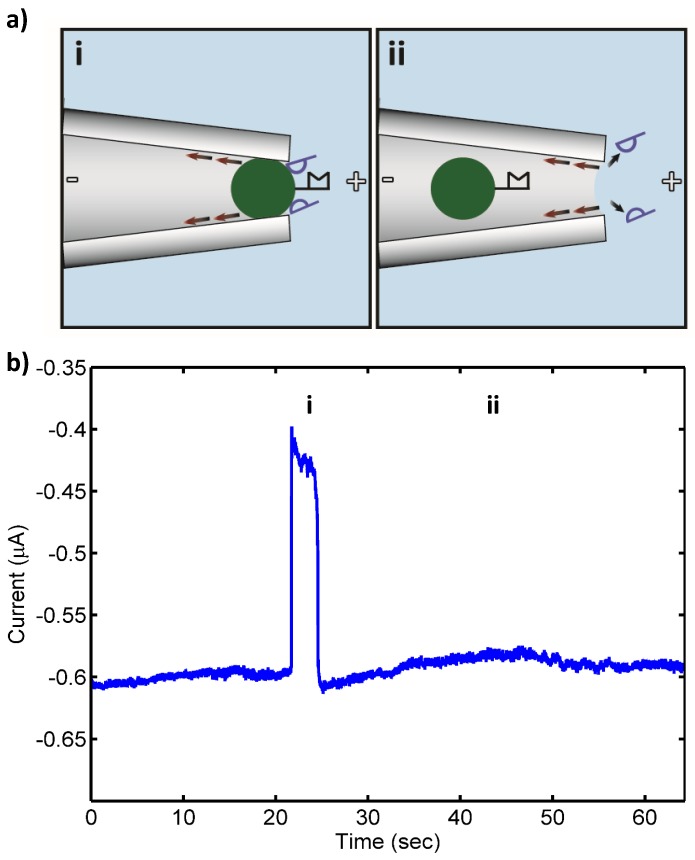
(**a**) Schematic of control (noncomplementary) *Pseudomonas putida* rRNA (blue, 10 pM) nonspecifically bound to bead-PNA probe conjugates under applied electric field in the drawn micropipette tip: (i) open pore state as bead with bound RNA approaches the micropipette tip (“pore”); (ii) transient blockade of the pore by the PNA-bead conjugate with nonspecifically bound noncomplementary RNA (blocked pore state); and (iii) relief of the pore blockade after the noncomplementary RNA of control bacteria was detached from the bead in the strong electric field at the micropipette tip and the bead is carried away from the pore by the opposing electroosmotic flow (red arrows) (open pore state). (**b**) Measured ionic current through the pore: (i) open pore current; (ii) current blockade by PNA-bead conjugates with bound RNA; and (iii) relief of the current blockade after the nonspecifically bound RNA was detached from the bead and the lack of tightly bound, complementary 16S rRNA resulted in the bead conjugate being swept from the pore by the opposing electroosmotic flow.

Target 16S rRNA at concentrations ranging from 10 pM to 100 fM was detected by the sensor with no false negatives. Also, only a few transient blocks (<60 s) were observed in control experiments that otherwise gave no false positives. Figure 1 shows a schematic of the pore blockage and the corresponding current measurements for target 16S rRNA at 10 pM, while Figure 2 shows a schematic of the transient pore blockage occasionally observed with non-complementary control 16S rRNA and the corresponding current trace for *P. putida* rRNA at 10 pM. Notably, target 16S rRNA at 10 fM was also detected in three separate experiments with no false negatives, and the controls incurred no false positives at this concentration. However, in the case of 1 fM target 16S rRNA, no detection events were observed with the detection probe or the universal probe. We concluded that the concentration detection limit of our sensor for 16S rRNA is ~10 fM. This limit is consistent with previous work with our sensor where we found a 10 fM LOD for 1613-base ssDNA, which corresponds to ~100–1000 CFU/mL (depending on the growth rate of the bacterium at the time of harvest and the corresponding rRNA levels within the cells) [24,25]. In the experiments reported here, we observed fewer transient blockades compared to previous studies with ssDNA detection. This might be due to the use of higher stringency hybridization conditions in this work. The high stringency hybridization conditions reduce the non-specific binding of non-complementary RNA to PNA-beads, therefore fewer beads with non-specifically bound RNA were able to reach the sensing zone and block the pore temporarily.

Future work will focus on 16S rRNA detection against a more realistic background of microbial nucleic acid. Work is also underway to scale-down the detector to the nano-regime in pursuit of still lower limits of detection.

## 4. Conclusions

Our previously described, PCR-free, optics-free detector for nucleic acids of specific sequence was used successfully to detect *E. coli* (ATCC 25922) 16S rRNA at 10 fM, which corresponds to ~100–1000 CFU/mL. The detector, as configured for *E. coli* 16S rRNA, did not give a signal in the presence of total RNA extracted from two control bacterial species, *Pseudomonas fluorescens* (ATCC 13525) and *Pseudomonas putida* (ATCC 12633) whose 16S rRNA genes are closely related to the target *E. coli*. To fully realize its potential, the detector would be integrated into a device containing a sample preparation frontend including cell lysis (e.g., chemical, electrical, mechanical or thermal), and DNA separation (e.g., silica-based surface affinity, electrostatic interaction, nanoporous membrane filtration, and functionalized microparticles), which have been shown to accomplish these tasks together in typically 10–15 min [33]. Although separation of RNA generally is more problematic due to its short lifetime and the ubiquitous presence of RNases, rapid methods (<15 min) to accomplish this task have also been reported [34]. Integrated together with these components, our detector shows promise for a low-cost, portable system for bacterial pathogen detection.

## Figures and Tables

**Table 1:** Summary of experiment (Expt.) results for target and control samples based on their 16S rRNA concentration. A positive result was defined as a blockade that persisted for >60 s and that was reversible by reversing the field polarity. A negative result was defined as the observation of either no block or a transient block (<60 s).

	Target *E. coli* (ATCC 25922)		Control Bacterium 1 (ATCC 12633)		Control Bacterium 2 (ATCC 13525)	
[16S rRNA]	Detection?	Positive control?	Detection?	Positive control?	Detection?	Positive control?
10 pM Expt. 1	Yes	Yes	No *	Yes	No	Yes
10 pM Expt. 2	Yes	Yes ^%^	No	Yes	No	Yes
10 pM Expt. 3	Yes	Yes	No	Yes	No *	Yes
1 pM	Yes	Yes	No	Yes	No	Yes
100 fM	Yes	Yes	No	Yes	No	Yes
10 fM Expt. 1	Yes	Yes	No	Yes	No	Yes
10 fM Expt. 2	Yes	Yes	No	Yes	No	Yes
10 fM Expt. 3	Yes	Yes	No	Yes	No	Yes
1 fM	No	No	No	No	No	No

* Transient block observed; ^%^ Transient block observed, followed by permanent block.

## Supplementary Materials

Bioanalyzer (Figure S-1) and blockade current data (Section A) are presented in the Supplementary Materials.
