# Supplementary material for: PCR-Independent Detection of Bacterial Species-Specific 16S rRNA at 10 fM by a Pore-Blockage Sensor
Source: Biosensors (Basel). 2016 Jul 22;6(3):37. doi: 10.3390/bios6030037 (PMC5039656; doi:10.3390/bios6030037)
Supplement: Supplementary file 1 [file biosensors-06-00037-s001.pdf]

Supplementary Materials: PCR-Independent Detection of Bacterial Species-Specific 16S rRNA at 10 fM by a Pore-Blockage Sensor

Leyla Esfandiari, Siqing Wang, Siqi Wang, Anisha Banda, Michael Lorenzini, Gayane Kocharyan, Harold G. Monbouquette and Jacob J. Schmidt

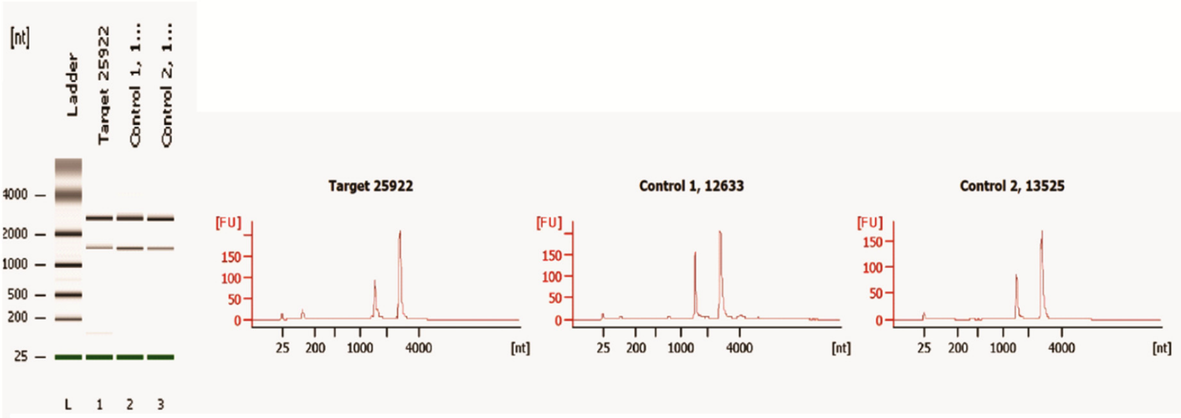

Figure S1. Chip−gel electrophoresis analysis of target and the two control bacteria. Fluorescence units versus RNA length was plotted for each sample. The first peak on each graph represents 16S rRNA and the second peak represents 23S rRNA. The ratio of 16rRNA to 23S rRNA is used by the instrument to calculate the integrity number.

A. Measurement results for Target and Control RNA from 10 pM to 1 fM

I_{open} is the current measured in the capillary with no obstruction, I_{block} is the current measured with an obstructing bead present, $R_{open} = 25\ V/I_{open}$, $\Delta R = R_{open} - V/I_{block}$. A Permanent block is noted if the block persisted beyond 60 s, and was reversible when the sign of the applied voltage was reversed.

10 pM Target

Table S1. Target detection Experiment 1.

	I_{open} (µA)	I_{block} (µA)	$(I_o - I_b)/I_o$	R_{open} (Ω)	ΔR (Ω)	ΔR/R	Block Duration (s)
	−0.576	−0.513	0.109	4.34 E+07	5.33 E+06	0.123	Permanent
	−0.58	−0.516	0.11	4.31 E+07	5.35 E+06	0.124	Permanent
	−0.589	−0.535	0.092	4.24 E+07	4.28 E+06	0.101	Permanent
	−0.588	−0.531	0.097	4.25 E+07	4.56 E+06	0.107	Permanent
	−0.59	−0.533	0.097	4.24 E+07	4.53 E+06	0.107	Permanent
Average	−0.585	−0.526	0.101	4.30 E+07	4.81 E+06	0.112	
Std. Dev.	0.006	0.009	0.007	4.19 E+05	4.41 E+05	0.009	

Table S2. Target positive control Experiment 1.

	I_{open} (μA)	I_{block} (μA)	$(I_o - I_b)/I_o$	R_{open} (Ω)	ΔR (Ω)	ΔR/R	Block Duration (s)
	−0.587	−0.498	0.152	4.26 E+07	7.61 E+06	0.179	Permanent
	−0.588	−0.481	0.182	4.25 E+07	9.46 E+06	0.222	Permanent
	−0.589	−0.501	0.149	4.24 E+07	7.46 E+06	0.176	Permanent
	−0.588	−0.515	0.124	4.25 E+07	6.03 E+06	0.142	Permanent
	−0.601	−0.504	0.161	4.159 E+07	8.01 E+06	0.192	Permanent
Average	−0.591	−0.500	0.154	4.15 E+07	7.71E+06	0.182	
Std. Dev.	0.005	0.011	0.019	3.78 E+05	1.10 E+05	0.026	

Table S3. Control Bacteria 1 detection Experiment 1.

I_{open} (μA)	I_{block} (μA)	$(I_o - I_b)/I_o$	R_{open} (Ω)	ΔR (Ω)	ΔR/R	Block Duration (s)
−0.595	−0.414	0.304	4.20 E+07	1.834 E+07	0.437	3

Table S4. Control Bacteria 1 positive control Experiment 1.

	I_{open} (μA)	I_{block} (μA)	$(I_o - I_b)/I_o$	R_{open} (Ω)	ΔR (Ω)	ΔR/R	Block Duration (s)
	−0.601	−0.497	0.173	4.16 E+07	8.70 E+06	0.209	Permanent
	−0.598	−0.501	0.162	4.18 E+07	8.09 E+06	0.193	Permanent
	−0.601	−0.498	0.171	4.16 E+07	8.60 E+06	0.207	Permanent
	−0.605	−0.502	0.170	4.13 E+07	8.48 E+06	0.205	Permanent
	−0.603	−0.500	0.171	4.15 E+07	8.54 E+06	0.206	Permanent
Average	−0.602	−0.499	0.170	4.17 E+07	8.48 E+06	0.204	
Std.Dev.	0.002	0.001	0.004	1.67 E+05	2.09 E+05	0.005	

Control Bacteria 2 detection Experiment 1

No capillary blockades detected

Table S5. Control Bacteria 2 positive control Experiment 1.

	I_{open} (μA)	I_{block} (μA)	$(I_o - I_b)/I_o$	R_{open} (Ω)	ΔR (Ω)	ΔR/R	Block Duration (s)
	−0.57	−0.478	0.161	4.39 E+07	8.44 E+06	0.192	Permanent
	−0.591	−0.502	0.151	4.23 E+07	7.50 E+06	0.177	Permanent
	−0.586	−0.497	0.152	4.27 E+07	7.64 E+06	0.179	Permanent
Average	−0.582	−0.492	0.155	4.29 E+07	7.86 E+06	0.183	
Std.Dev.	0.009	0.010	0.005	6.66 E+05	4.15 E+05	0.007	

Table S6. Target detection Experiment 2.

	I_{open} (μA)	I_{block} (μA)	$(I_o - I_b)/I_o$	R_{open} (Ω)	ΔR (Ω)	ΔR/R	Block Duration (s)
	−0.6	−0.521	0.131	4.16 E+07	6.31 E+06	0.151	Permanent
	−0.589	−0.531	0.098	4.24 E+07	4.63 E+06	0.109	Permanent
	−0.589	−0.535	0.091	4.24 E+07	4.28 E+06	0.100	Permanent
	−0.593	−0.527	0.111	4.21 E+07	5.27 E+06	0.125	Permanent
	−0.613	−0.533	0.130	4.07 E+07	6.12 E+06	0.150	Permanent
Average	−0.597	−0.529	0.112	4.21 E+07	5.32 E+06	0.127	
Std.Dev.	0.009	0.005	0.016	6.37 E+06	7.97 E+05	0.020	

Table S7. Target positive control Experiment 2.

	I_{open} (μA)	I_{block} (μA)	$(I_o - I_b)/I_o$	R_{open} (Ω)	ΔR (Ω)	ΔR/R	Block Duration (s)
	−0.65	−0.524	0.193	3.84 E+07	9.24 E+06	0.240	Permanent
	−0.632	−0.533	0.156	3.95 E+07	7.34 E+06	0.185	Permanent
	−0.645	−0.535	0.170	3.87 E+07	7.96 E+06	0.205	4 s
	−0.637	−0.547	0.141	3.92 E+07	6.45 E+06	0.164	Permanent
	−0.645	−0.533	0.173	3.87 E+07	8.14 E+06	0.210	Permanent
Average	−0.642	−0.534	0.167	3.89 E+07	7.83 E+06	0.201	
Std.Dev.	0.006	0.007	0.017	3.91 E+05	9.21 E+05	0.025	

Control Bacteria 1 detection Experiment 1

No capillary blockades detected

Table S8. Control Bacteria 1 positive control Experiment 2.

	I_{open} (μA)	I_{block} (μA)	$(I_o - I_b)/I_o$	R_{open} (Ω)	ΔR (Ω)	ΔR/R	Block Duration (s)
	−0.576	−0.514	0.107	4.34 E+07	5.23 E+06	0.120	Permanent
	−0.582	−0.513	0.118	4.29 E+07	5.77 E+06	0.134	Permanent
	−0.579	−0.5	0.136	4.31 E+07	6.82 E+06	0.158	Permanent
	−0.577	−0.515	0.107	4.33 E+07	5.21 E+06	0.120	Permanent
	−0.581	−0.513	0.117	4.30 E+07	5.70 E+06	0.132	Permanent
Average	−0.579	−0.511	0.117	4.31 E+07	5.75 E+06	0.133	
Std.Dev.	0.002	0.005	0.010	1.70 E+05	5.83 E+05	0.013	

Control Bacteria 2 detection Experiment 2

No capillary blockades detected

Table S9. Control Bacteria 2 positive control Experiment 2.

	I_{open} (μA)	I_{block} (μA)	$(I_o - I_b)/I_o$	R_{open} (Ω)	ΔR (Ω)	ΔR/R	Block Duration (s)
	−0.56	−0.514	0.082	4.46 E+07	3.99 E+06	0.089	Permanent
	−0.562	−0.513	0.087	4.44 E+07	4.24 E+06	0.095	Permanent
	−0.559	−0.513	0.082	4.47 E+07	4.01 E+06	0.089	Permanent
	−0.561	−0.515	0.081	4.45 E+07	3.98 E+06	0.089	Permanent
Average	−0.561	−0.514	0.083	4.46 E+07	4.05 E+06	0.090	
Std.Dev.	0.001	0.001	0.002	8.91 E+04	1.10 E+05	0.002	

Table S10. Target bacteria positive control Experiment 3.

	I_{open} (μA)	I_{block} (μA)	$(I_o - I_b)/I_o$	R_{open} (Ω)	ΔR (Ω)	ΔR/R	Block Duration (s)
	−0.61	−0.503	0.175	4.09 E+07	8.71 E+06	0.212	Permanent
	−0.58	−0.495	0.146	4.31 E+07	7.40 E+06	0.171	Permanent
	−0.597	−0.511	0.144	4.18 E+07	7.04 E+06	0.168	Permanent
	−0.6	−0.521	0.131	4.16 E+07	6.31 E+06	0.151	Permanent
	−0.59	−0.5	0.152	4.23 E+07	7.62 E+06	0.18	Permanent
Average	−0.595	−0.506	0.150	4.19 E+07	7.42 E+06	0.176	
Std.Dev.	0.010	0.009	0.014	7.09 E+05	7.85 E+05	0.020	

Table S11. Target bacteria detection Experiment 3.

	I_{open} (μA)	I_{block} (μA)	$(I_o - I_b)/I_o$	R_{open} (Ω)	ΔR (Ω)	ΔR/R	Block Duration (s)
	−0.61	−0.503	0.175	4.09 E+07	8.71 E+06	0.212	Permanent
	−0.595	−0.485	0.184	4.20 E+07	9.52 E+06	0.226	Permanent
	−0.598	−0.513	0.142	4.18 E+07	6.92 E+06	0.165	Permanent
	−0.580	−0.418	0.279	4.31 E+07	1.67 E+07	0.388	Permanent
Average	−0.595	−0.480	0.195	4.20 E+07	1.04 E+07	0.248	
Std.Dev.	0.012	0.043	0.059	8.73 E+05	4.30 E+06	0.097	

Control Bacteria 1 detection Experiment 3

No capillary blockades detected

Table S12. Control Bacteria 1 positive control Experiment 3.

	I_{open} (μA)	I_{block} (μA)	$(I_o - I_b)/I_o$	R_{open} (Ω)	ΔR (Ω)	ΔR/R	Block Duration (s)
	−0.58	−0.501	0.136	4.31 E+07	6.79 E+06	0.157	Permanent
	−0.595	−0.521	0.124	4.20 E+07	5.96 E+06	0.142	Permanent
	−0.599	−0.513	0.143	4.17 E+07	6.99 E+06	0.167	Permanent
Average	−0.591	−0.511	0.134	4.22 E+07	6.58 E+06	0.155	
Std.Dev.	0.008	0.008	0.007	5.89 E+05	4.45 E+05	0.010	

Table S13. Control Bacteria 2 detection Experiment 3.

	I_{open} (μA)	I_{block} (μA)	$(I_o - I_b)/I_o$	R_{open} (Ω)	ΔR (Ω)	ΔR/R	Block Duration (s)
	−0.612	−0.534	0.127	4.08 E+07	5.96 E+06	0.146	4 s
	−0.614	−0.521	0.151	4.07 E+07	7.26 E+06	0.178	3 s
Average	−0.613	−0.528	0.139	4.07 E+07	6.61 E+06	0.162	
Std.Dev.	0.001	0.007	0.012	6.65 E+04	6.50 E+05	0.016	

Table S14. Control Bacteria 2 positive control Experiment 3.

	I_{open} (μA)	I_{block} (μA)	$(I_o - I_b)/I_o$	R_{open} (Ω)	ΔR (Ω)	ΔR/R	Block Duration (s)
	−0.5786	−0.513	0.113	4.32 E+07	5.52 E+06	0.127	Permanent
	−0.58	−0.518	0.106	4.31 E+07	5.15 E+06	0.119	Permanent
	−0.6	−0.535	0.108	4.16 E+07	5.06 E+06	0.121	Permanent
	−0.598	−0.531	0.112	4.18 E+07	5.27 E+06	0.126	Permanent
	−0.595	−0.533	0.104	4.20 E+07	4.88 E+06	0.116	Permanent
Average	−0.590	−0.526	0.108	4.26 E+07	5.18 E+06	0.122	
Std.Dev.	0.009	0.008	0.003	6.71 E+05	2.13 E+05	0.004	

1 pM Target

Table S15. Target bacteria detection experiment.

	I_{open} (μA)	I_{block} (μA)	$(I_o - I_b)/I_o$	R_{open} (Ω)	ΔR (Ω)	ΔR/R	Block Duration (s)
	−0.61	−0.503	0.175	4.09 E+07	8.71 E+06	0.212	Permanent
	−0.621	−0.518	0.165	4.02 E+07	8.00 E+06	0.198	Permanent
	−0.612	−0.545	0.109	4.08 E+07	5.02 E+06	0.122	Permanent
	−0.609	−0.531	0.128	4.10 E+07	6.03 E+06	0.146	Permanent
	−0.595	−0.532	0.105	4.20 E+07	4.97 E+06	0.118	Permanent
Average	−0.609	−0.525	0.136	4.06 E+07	6.55 E+06	0.159	
Std.Dev.	0.008	0.014	0.028	5.82 E+05	1.54 E+06	0.038	

Table S16. Target bacteria positive control experiment.

	I_{open} (μA)	I_{block} (μA)	$(I_o - I_b)/I_o$	R_{open} (Ω)	ΔR (Ω)	ΔR/R	Block Duration (s)
	−0.587	−0.5	0.148	4.25 E+07	7.41 E+06	0.174	Permanent
	−0.598	−0.502	0.160	4.18 E+07	7.99 E+06	0.191	Permanent
	−0.6	−0.521	0.131	4.16 E+07	6.31 E+06	0.151	Permanent
	−0.604	−0.53	0.122	4.13 E+07	5.77 E+06	0.139	Permanent
Average	−0.597	−0.513	0.140	4.20 E+07	6.87 E+06	0.164	
Std.Dev.	0.006	0.012	0.014	4.50 E+05	8.73 E+05	0.019	

Control Bacteria 1 detection experiment

No capillary blockades detected

Table S17. Control Bacteria 1 positive control experiment.

	I_{open} (μA)	I_{block} (μA)	$(I_o - I_b)/I_o$	R_{open} (Ω)	ΔR (Ω)	ΔR/R	Block Duration (s)
	−0.612	−0.512	0.163	4.08 E+07	7.97 E+06	0.195	Permanent
	−0.623	−0.532	0.146	4.01 E+07	6.86 E+06	0.171	Permanent
	−0.611	−0.524	0.142	4.09 E+07	6.79 E+06	0.166	Permanent
	−0.625	−0.532	0.148	4.00 E+07	6.99 E+06	0.174	Permanent
Average	−0.618	−0.525	0.150	4.06 E+07	7.15 E+06	0.176	
Std.Dev.	0.006	0.008	0.007	4.18 E+05	4.79 E+05	0.011	

Control Bacteria 2 detection experiment

No capillary blockades detected

Table S18. Control Bacteria 2 positive control experiment.

	I_{open} (μA)	I_{block} (μA)	$(I_o - I_b)/I_o$	R_{open} (Ω)	ΔR (Ω)	ΔR/R	Block Duration (s)
	−0.588	−0.502	0.146	4.25 E+07	7.28 E+06	0.171	Permanent
	−0.613	−0.521	0.150	4.07 E+07	7.20 E+06	0.176	Permanent
	−0.611	−0.534	0.126	4.09 E+07	5.89 E+06	0.144	Permanent
	−0.587	−0.511	0.129	4.25 E+07	6.33 E+06	0.148	Permanent
	−0.597	−0.5	0.162	4.18 E+07	8.12 E+06	0.194	Permanent
Average	−0.599	−0.513	0.142	4.14 E+07	6.96 E+06	0.1665	
Std.Dev.	0.011	0.012	0.013	7.78 E+05	7.78 E+06	0.018	

100 fM Target

Table S19. Target bacteria detection experiment.

	I_{open} (μA)	I_{block} (μA)	$(I_o - I_b)/I_o$	R_{open} (Ω)	ΔR (Ω)	ΔR/R	Block Duration (s)
	−0.589	−0.5	0.151	4.24 E+07	7.55 E+06	0.178	Permanent
	−0.599	−0.512	0.145	4.17 E+07	7.09 E+06	0.169	Permanent
	−0.6	−0.554	0.076	4.16 E+07	3.45 E+06	0.083	Permanent
	−0.597	−0.515	0.137	4.18 E+07	6.66 E+06	0.159	Permanent
	−0.597	−0.503	0.157	4.18 E+07	7.82 E+06	0.186	Permanent
Average	−0.596	−0.517	0.133	4.19 E+07	6.52 E+06	0.159	
Std.Dev.	0.003	0.019	0.0292	2.74 E+07	1.58 E+06	0.037	

Table S20. Target bacteria positive control experiment.

	I_{open} (μA)	I_{block} (μA)	$(I_o - I_b)/I_o$	R_{open} (Ω)	ΔR (Ω)	ΔR/R	Block Duration (s)
	−0.623	−0.532	0.146	4.01 E+07	6.86 E+06	0.178	Permanent
	−0.615	−0.487	0.208	4.07 E+07	1.07 E+07	0.169	Permanent
	−0.596	−0.453	0.240	4.19 E+07	1.32 E+07	0.083	Permanent
	−0.604	−0.421	0.303	4.14 E+07	1.80 E+07	0.159	Permanent
Average	−0.610	−0.473	0.224	4.10 E+07	1.22 E+07	0.296	
Std.Dev.	0.012	0.048	0.065	8.01 E+05	4.67 E+06	0.110	

Control Bacteria 1 detection experiment

No capillary blockades detected

Table S21. Control Bacteria 1 positive control experiment.

	I_{open} (μA)	I_{block} (μA)	$(I_o - I_b)/I_o$	R_{open} (Ω)	ΔR (Ω)	ΔR/R	Block Duration (s)
	−0.594	−0.52	0.124	4.2 E+07	5.98 E+06	0.142	Permanent
	−0.591	−0.521	0.118	4.23 E+07	5.68 E+06	0.134	Permanent
	−0.597	−0.543	0.090	4.18 E+07	4.16 E+06	0.0994	Permanent
	−0.594	−0.518	0.127	4.20 E+07	6.17 E+06	0.146	Permanent
	−0.61	−0.521	0.145	4.09 E+07	7.00 E+06	0.170	Permanent
Average	−0.597	−0.525	0.121	4.20 E+07	5.80 E+06	0.138	
Std.Dev.	0.006	0.009	0.0179	4.70 E+05	9.28 E+05	0.023	

Control Bacteria 2 detection experiment

No capillary blockades detected

Table S22. Control Bacteria 2 positive control experiment.

	I_{open} (μA)	I_{block} (μA)	$(I_o - I_b)/I_o$	R_{open} (Ω)	ΔR (Ω)	ΔR/R	Block Duration (s)
	−0.613	−0.532	0.132	4.07 E+07	6.20 E+06	0.152	Permanent
	−0.632	−0.525	0.169	3.95 E+07	8.06 E+06	0.203	Permanent
	−0.626	−0.513	0.180	3.99 E+07	8.79 E+06	0.220	Permanent
	−0.615	−0.528	0.141	4.06 E+07	6.69 E+06	0.164	Permanent
	−0.618	−0.512	0.171	4.04 E+07	8.37 E+06	0.207	Permanent
Average	−0.621	−0.522	0.158	4.00 E+07	7.62 E+06	0.189	
Std.Dev.	0.007	0.008	0.018	4.66 E+05	9.98 E+05	0.026	

<u>10 fM Target</u>

Table S23. Target bacteria detection Eperiment 1.

	I_{open} (μA)	I_{block} (μA)	$(I_o - I_b)/I_o$	R_{open} (Ω)	ΔR (Ω)	ΔR/R	Block Duration (s)
	−0.587	−0.522	0.110	4,25 E+07	5.30 E+06	0.124	Permanent
	−0.593	−0.526	0.112	4.21 E+07	5.37 E+06	0.127	Permanent
	−0.585	−0.513	0.1230	4.27 E+07	5.99 E+06	0.140	Permanent
	−0.601	−0.516	0.1414	4.15 E+07	6.85 E+06	0.164	Permanent
	−0.612	−0.528	0.137	4.08 E+07	6.49 E+06	0.159	Permanent
Average	−0.596	−0.521	0.1250	4.24 E+07	6.00 E+06	0.143	
Std.Dev.	0.009	0.005	0.0124	7.22 E+05	6.09 E+05	0.0162	

Table S24. Target bacteria positive control Experiment 1.

	I_{open} (μA)	I_{block} (μA)	$(I_o - I_b)/I_o$	R_{open} (Ω)	ΔR (Ω)	ΔR/R	Block Duration (s)
	−0.612	−0.512	0.163	4.08 E+07	7.97 E+06	0.195	Permanent
	−0.611	−0.524	0.142	4.09 E+07	6.79 E+06	0.166	Permanent
	−0.632	−0.516	0.183	3.95 E+07	8.89 E+06	0.224	Permanent
	−0.614	−0.519	0.154	4.07 E+07	7.45 E+06	0.183	Permanent
	−0.626	−0.527	0.158	3.99 E+07	7.50 E+06	0.187	Permanent
Average	−0.619	−0.520	0.160	4.04 E+07	7.72 E+06	0.191	
Std.Dev.	0.008	0.005	0.013	5.47 E+05	6.95 E+05	0.019	

Control Bacteria 1 detection experiment

No capillary blockades detected

Table S25. Control Bacteria 1 positive control Experiment 1.

	I_{open} (μA)	I_{block} (μA)	$(I_o - I_b)/I_o$	R_{open} (Ω)	ΔR (Ω)	ΔR/R	Block Duration (s)
	−0.621	−0.513	0.173	4.02 E+07	8.47 E+06	0.210	Permanent
	−0.623	−0.514	0.174	4.01 E+07	8.50 E+06	0.212	Permanent
	−0.611	−0.527	0.137	4.09 E+07	6.52 E+06	0.159	Permanent
	−0.651	−0.514	0.210	3.84 E+07	1.02 E+06	0.266	Permanent
	−0.648	−0.519	0.199	3.85 E+07	9.58 E+06	0.248	Permanent
Average	−0.631	−0.517	0.179	4.04 E+07	8.66 E+06	0.219	
Std.Dev.	0.015	0.005	0.025	1.03 E+06	1.26 E+06	0.036	

Control Bacteria 2 detection experiment

No capillary blockades detected

Table S26. Control Bacteria 2 positive control Experiment 1.

	I_{open} (μA)	I_{block} (μA)	$(I_o - I_b)/I_o$	R_{open} (Ω)	ΔR (Ω)	ΔR/R	Block Duration (s)
	−0.612	−0.523	0.145	4,08 E+07	6.95 E+06	0.170	Permanent
	−0.598	−0.512	0.143	4.18 E+07	7.02 E+06	0.167	Permanent
	−0.613	−0.572	0.066	4.07 E+07	2.92 E+06	0.0716	Permanent
	−0.592	−0.517	0.126	4.22 E+07	6.12 E+06	0.145	Permanent
	−0.595	−0.51	0.142	4.20 E+07	7.00 E+06	0.166	Permanent
Average	−0.602	−0.527	0.125	4.11 E+07	6.00 E+06	0.144	
Std.Dev.	0.008	0.023	0.029	6.24 E+05	1.57 E+06	0.037	

Table S27. Target bacteria detection Experiment 2.

	I_{open} (μA)	I_{block} (μA)	$(I_o - I_b)/I_o$	R_{open} (Ω)	ΔR (Ω)	ΔR/R	Block Duration (s)
	−0.601	−0.503	0.163	4.15 E+07	8.10 E+06	0.194	Permanent
	−0.613	−0.512	0.164	4.07 E+07	8.04 E+06	0.197	Permanent
	−0.604	−0.532	0.119	4.13 E+07	5.60 E+06	0.135	Permanent
	−0.615	−0.501	0.185	4.06 E+07	9.24 E+06	0.227	Permanent
	−0.624	−0.532	0.147	4.00 E+07	6.92 E+06	0.172	Permanent
Average	−0.611	−0.516	0.155	4.12 E+07	7.58 E+06	0.185	
Std.Dev.	0.008	0.013	0.021	5.67 E+05	1.23 E+06	0.030	

Table S28. Target bacteria positive control Experiment 2.

	I_{open} (μA)	I_{block} (μA)	$(I_o - I_b)/I_o$	R_{open} (Ω)	ΔR (Ω)	ΔR/R	Block Duration (s)
	−0.623	−0.489	0.215	4.01 E+07	1.09 E+07	0.274	Permanent
	−0.643	−0.482	0.250	3.89 E+07	1.30 E+07	0.334	Permanent
	−0.632	−0.453	0.283	3.96 E+07	1.56 E+07	0.395	Permanent
	−0.611	−0.474	0.224	4.09 E+07	1.18 E+07	0.289	Permanent
	−0.621	−0.411	0.338	4.03 E+07	2.06 E+07	0.511	Permanent
Average	−0.626	−0.462	0.262	4.00 E+07	1.44 E+07	0.361	
Std.Dev.	0.012	0.031	0.050	7.68 E+05	3.87 E+06	0.096	

Control Bacteria 1 detection Experiment 2

No capillary blockades detected

Table S29. Control Bacteria 1 positive control Experiment 2.

	I_{open} (μA)	I_{block} (μA)	$(I_o - I_b)/I_o$	R_{open} (Ω)	ΔR (Ω)	ΔR/R	Block Duration (s)
	−0.621	−0.513	0.173	4.02 E+07	8.47 E+06	0.210	Permanent
	−0.633	−0.522	0.175	3.94 E+07	8.39 E+06	0.212	Permanent
	−0.634	−0.523	0.175	3.94 E+07	8.36 E+06	0.212	Permanent
	−0.615	−0.512	0.167	4.06 E+07	8.17 E+06	0.201	Permanent
	−0.614	−0.511	0.167	4.07 E+07	8.20 E+06	0.201	Permanent
Average	−0.623	−0.516	0.171	3.97 E+07	8.32 E+06	0.207	
Std.Dev.	0.008	0.005	0.003	5.73 E+05	1.14 E+05	0.005	

Control Bacteria 2 detection Experiment 2

No capillary blockades detected

Table S30. Control Bacteria 2 positive control Experiment 2.

	I_{open} (μA)	I_{block} (μA)	$(I_o - I_b)/I_o$	R_{open} (Ω)	ΔR (Ω)	ΔR/R	Block Duration (s)
	−0.61	−0.501	0.178	4.09 E+07	8.91 E+06	0.217	Permanent
	−0.611	−0.512	0.162	4.09 E+07	7.91 E+06	0.193	Permanent
	−0.611	−0.513	0.160	4.09 E+07	7.82 E+06	0.191	Permanent
	−0.613	−0.512	0.164	4.07 E+07	8.04 E+06	0.197	Permanent
	−0.618	−0.511	0.173	4.04 E+07	8.47 E+06	0.209	Permanent
Average	−0.613	−0.510	0.167	4.09 E+07	8.23 E+06	0.201	
Std.Dev.	0.002	0.004	0.006	1.97 E+05	4.08 E+05	0.010	

Table S31. Target bacteria detection Experiment 3.

	I_{open} (μA)	I_{block} (μA)	$(I_o - I_b)/I_o$	R_{open} (Ω)	ΔR (Ω)	ΔR/R	Block Duration (s)
	−0.543	−0.498	0.082	4.60 E+07	4.16 E+06	0.090	Permanent
	−0.555	−0.465	0.162	4.50 E+07	8.71 E+06	0.193	Permanent
	−0.542	−0.487	0.101	4.61 E+07	5.20 E+06	0.112	Permanent
	−0.574	−0.423	0.263	4.35 E+07	1.55 E+06	0.356	Permanent
	−0.534	−0.457	0.144	4.68 E+07	7.88 E+06	0.168	Permanent
Average	−0.550	−0.466	0.150	4.57 E+07	8.30 E+06	0.184	
Std.Dev.	0.0139	0.026	0.062	1.13 E+06	3.98 E+06	0.093	

Table S32. Target bacteria positive control Experiment 3.

	I_{open} (μA)	I_{block} (μA)	$(I_o - I_b)/I_o$	R_{open} (Ω)	ΔR (Ω)	ΔR/R	Block Duration (s)
	−0.588	−0.413	0.298	4.25 E+07	1.80 E+07	0.424	Permanent
	−0.579	−0.402	0.306	4.32 E+07	1.90 E+07	0.440	Permanent
	−0.582	−0.421	0.277	4.30 E+07	1.64 E+07	0.382	Permanent
Average	−0.583	−0.412	0.293	4.29 E+07	1.78 E+07	0.415	
Std.Dev.	0.005	0.010	0.015	3.36 E+05	1.30 E+06	0.030	

Control Bacteria 1 detection Experiment 3

No capillary blockades detected

Table S33. Control Bacteria 1 positive control Experiment 3.

	I_{open} (μA)	I_{block} (μA)	$(I_o - I_b)/I_o$	R_{open} (Ω)	ΔR (Ω)	ΔR/R	Block Duration (s)
	−0.556	−0.421	0.242	4.49 E+07	1.44 E+07	0.320	Permanent
	−0.561	−0.465	0.171	4.45 E+07	9.20 E+06	0.206	Permanent
	−0.564	−0.416	0.262	4.43 E+07	1.57 E+07	0.355	Permanent
	−0.558	−0.453	0.188	4.48 E+07	1.03 E+07	0.231	Permanent
	−0.56	−0.444	0.207	4.46 E+07	1.16 E+07	0.261	Permanent
Average	−0.560	−0.440	0.214	4.46 E+07	1.22 E+07	0.275	
Std.Dev.	0.002	0.018	0.033	2.16 E+05	2.45 E+06	0.055	

Control Bacteria 2 detection Experiment 3

No capillary blockades detected

1 fM Target

In the case of 1 fM, no current blockades were observed.

© 2016 by the authors. Submitted for possible open access publication under the terms and conditions of the Creative Commons Attribution (CC-BY) license (http://creativecommons.org/licenses/by/4.0/).
